# Outbreaks of Human *Salmonella* Infections Associated with Live Poultry, United States, 1990–2014

**DOI:** 10.3201/eid2210.150765

**Published:** 2016-10

**Authors:** Colin Basler, Thai-An Nguyen, Tara C. Anderson, Thane Hancock, Casey Barton Behravesh

**Affiliations:** Centers for Disease Control and Prevention, Atlanta, Georgia, USA

**Keywords:** zoonoses, poultry, animal, Salmonella, infection, salmonellosis, bacteria, outbreak, One Health, chicks, ducklings, goslings, poults, Easter, backyard, hatcheries

## Abstract

Backyard poultry flocks have increased in popularity concurrent with an increase in live poultry–associated salmonellosis (LPAS) outbreaks. Better understanding of practices that contribute to this emerging public health issue is needed. We reviewed outbreak reports to describe the epidemiology of LPAS outbreaks in the United States, examine changes in trends, and inform prevention campaigns. LPAS outbreaks were defined as ≥2 culture-confirmed human *Salmonella* infections linked to live poultry contact. Outbreak data were obtained through multiple databases and a literature review. During 1990–2014, a total of 53 LPAS outbreaks were documented, involving 2,630 illnesses, 387 hospitalizations, and 5 deaths. Median patient age was 9 years (range <1 to 92 years). Chick and duckling exposure were reported by 85% and 38% of case-patients, respectively. High-risk practices included keeping poultry inside households (46% of case-patients) and kissing birds (13%). Comprehensive One Health strategies are needed to prevent illnesses associated with live poultry.

## Abstract 2

These outbreaks underscore the need for a comprehensive One Health approach that integrates human, animal, and environmental health.

*Salmonella* species are zoonotic bacteria found in the intestinal tract of many animals, including cattle, pigs, horses, other mammals, reptiles, amphibians, and poultry (e.g., chickens, ducks, geese, and turkeys) (*1*). Nontyphoidal salmonellosis causes an estimated 1.2 million illnesses, 23,000 hospitalizations, and 450 deaths annually in the United States (*2*). *Salmonella* infection typically manifests as acute gastroenteritis that develops 12–72 hours after exposure. Young children, persons >65 years of age, and immunocompromised persons are at greater risk for serious complications, including septicemia, joint or brain infections, and death (*3*).

Although *Salmonella* is commonly transmitted through food, recent outbreaks have highlighted direct or indirect contact with animals as a frequent route of transmission (*4*). An estimated 11% of all *Salmonella* infections are attributed to animal exposure annually, with the highest rates of illness and death occurring among children (*1*). Since 2007, numerous outbreaks of human *Salmonella* infections linked to contact with animals and their environments have been investigated, including those involving contact with turtles, bearded dragons, African dwarf frogs, hedgehogs, and backyard poultry (*5*). Poultry can be persistent subclinical shedders and can appear healthy while shedding *Salmonella* bacteria (*6*). Zoonotic salmonellosis outbreak investigations require a One Health approach because they occur at the intersection of human and animal health (*7*).

In the United States, live poultry–associated salmonellosis (LPAS) outbreaks have been documented since 1955 (*8*). Historically, these outbreaks involved young children, occurred in the spring months around Easter, and were associated with birds obtained as pets (*9*). Baby poultry were often dyed bright colors, making them more attractive to young children. Currently, public health officials are identifying LPAS outbreaks linked to backyard poultry flocks that are affecting adults and children. Most of these outbreaks begin in the spring but continue over many months. The first multistate outbreak where the association with backyard flocks was recognized occurred in 2007 (*10*). Since that time, the popularity of backyard flocks has increased substantially (*11*). Most chicks sold for backyard flocks are produced by a network of mail-order hatcheries (*9*). Disease control guidance for hatcheries is provided by the US Department of Agriculture National Poultry Improvement Plan, which is a voluntary state, federal, and industry cooperative program aimed at eliminating certain diseases from poultry breeding flocks and hatcheries (*12*). We reviewed outbreak reports from 1990–2014 to describe the epidemiology of LPAS outbreaks in the United States, to identify changes in trends, and to identify practices of concern among case-patients to better inform future prevention campaigns.

## Methods

We defined LPAS outbreaks as >2 culture-confirmed human *Salmonella* infections in the United States with a combination of epidemiologic, laboratory, or traceback evidence linking illnesses to live poultry contact. Data sources included PulseNet, the national molecular subtyping network for foodborne disease surveillance in the United States; the Centers for Disease Control and Prevention (CDC) Outbreak Response and Prevention Branch’s outbreak management database; and CDC’s National Outbreak Reporting System (*13*–*15*). Through these data sources, we collected outbreak summaries from 50 states and 4 US territories. Additionally, we conducted a literature review to identify any additional LPAS outbreaks that had not been reported to CDC. In January 2015, we searched PubMed without date or language restrictions and used combinations of the terms “salmonella,” “salmonellosis,” “outbreak,” “poultry,” and “United States.” To avoid including duplicate reports, we further reviewed outbreaks that occurred in the same year and for which identical *Salmonella* serotypes were reported.

A standardized live poultry exposure questionnaire was developed by officials at CDC, state and local health departments, and the National Poultry Improvement Plan. The questionnaire focused on patient demographics, baby and adult poultry contact, poultry purchases, flock management, and *Salmonella* awareness. The questionnaire was created in 2008, and since its creation, it has been administered to case-patients (or their parents/guardians) who were part of 21 multistate outbreak investigations during 2008–2013. To identify common patient characteristics and practices that might have increased the risk for *Salmonella* transmission from poultry to humans, we analyzed the results of these questionnaires by using SAS version 9.2 (SAS Institute, Cary, NC, USA).

## Results

**Figure 1:**
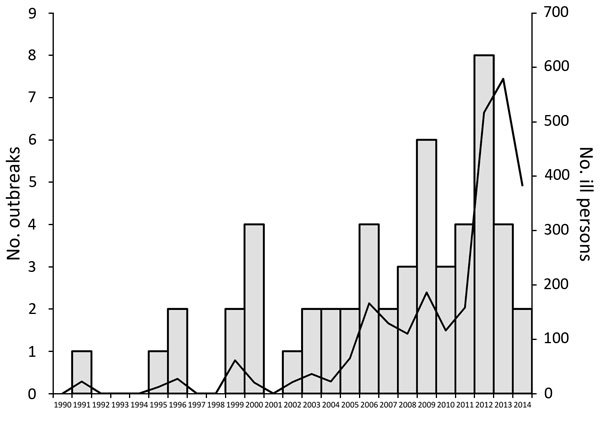
Number of live poultry–associated salmonellosis outbreaks and number of ill persons reported, by year, United States, 1991–2014.

A total of 53 LPAS outbreaks were documented in the United States during 1990–2014 (Table 1); these 53 outbreaks were associated with 2,630 illnesses, 387 hospitalizations, and 5 deaths (Figure 1). Median outbreak size was 26 case-patients (range 4–363 case-patients). Approximately 77% (41/53) of outbreaks were multistate outbreaks. 

**Table 1:** Details on live poultry–associated salmonellosis outbreaks, by year, United States, 1990–2014

Year	Serotype	No. illnesses	No. hospitalizations	No. deaths	Month of first illness in outbreak	Outbreak duration, mo	Reference(s)
1991	Hadar	22	4	0	April	2	(*16*)
1995	Montevideo	12	3	0	April	2	(*17*)
1996	Montevideo	11	0	0	April	2	(*17*)
1996	Montevideo	16	2	0	March	4	(*17*)
1999	Infantis	21	3	0	April	2	(*18*,*19*)
1999	Typhimurium	40	3	0	April	2	(*18*)
2000	Infantis	5	2	0	May	1	(*19*)
2000	Montevideo	4	0	0	May	2	
2000	Agona	4	0	0	February		
2000	Montevideo	7	0	0			
2002	Montevideo	21	0	0	March	2	
2003	Thompson	31	4	0	May	2	
2003	Unknown	5	0	0			
2004	Montevideo	4	0	0	March	2	
2004	Typhimurium	18	0	0	March		
2005	Montevideo	53	6	0	April		(*20*)
2005	Ohio	12	0	0			
2006	Typhimurium	14	7	0	May		
2006	I 4,[5],12:i:-	64	7	0	April	6	(*21*)
2006	Montevideo	84	8	0			(*20*,*21*)
2006	Ohio	4	1	0			(*21*)
2007	Montevideo	64	8	0	February	11	(*20*)
2007	Montevideo	65	3	0	March	7	(*22*)
2008	Kiambu	32	0	0	March	4	
2008	Montevideo	12	4	0			(*20*)
2008	Montevideo	66					
2009	Montevideo	96	16	1	January	12	(*20*)
2009	Johannesburg	7	2	0	May	1	(*23*)
2009	Thompson	26	1	0	February	6	(*22*)
2009	Typhimurium	36	7	0	May	4	(*23*,*24*)
2009	Pomona	6	0	0	March		
2009	Montevideo	15	1	0			(*23*)
2010	Typhimurium	54	0	0	May	4	
2010	Montevideo	55	7	0	February	4	(*20*)
2010	Braenderup	7		0	February		
2011	Johannesburg, Altona	96	20	0	February		(*25*,*26*)
2011	Berta	9	1	0	April	3	
2011	Hadar	25	2	0	March	5	
2011	Montevideo	28	2	0	March		(*20*)
2012	Infantis	54	4	0	February	8	
2012	Braenderup	48	6	0	July	6	
2012	Infantis	27	7	0	April	5	
2012	Muenchen	21	1	0	March	6	
2012	Hadar	46	13	0	March	5	(*27*)
2012	Montevideo	93	21	1	February	7	(*28*)
2012	Infantis, Newport, Lille, Thompson	195	34	3	March	6	(*29*,*30*)
2012	Thompson	33	4	0	February	8	
2013	Braenderup	53	1	0	March	5	
2013	Infantis, Mbandaka, Lille, Newport	158	29	0	March	7	(*31*–*33*)
2013	Montevideo	12	0	0	April		
2013	Typhimurium	356	62	0	March	7	(*33*)
2014	Infantis, Newport, Hadar	363	76	0	February	9	
2014	Typhimurium	20	5	0	March	7	
Total		2,630	387	5	March (median)	4.9 mo (median)	

The number of LPAS outbreaks reported annually has increased substantially in recent years (Figure 1). During 1990–2005, a total of 17 outbreaks were documented (1.06/year), with a median size of 12 case-patients per outbreak (range 4–53 case-patients). In comparison, during 2006–2014, a total of 36 outbreaks were documented (4/year), with a median size of 41 case-patients per outbreak (range 4–363 case-patients). The number of LPAS outbreaks peaked in 2012, with a total of 8 individual outbreaks. The 4 largest outbreaks occurred during 2012–2014. 

Reported outbreak onset dates ranged from January to July. Most (80%) outbreaks began in the months of February, March, and April. Outbreak duration ranged from 1 to 12 months, with an average duration of 4.9 months. 

**Figure 2:**
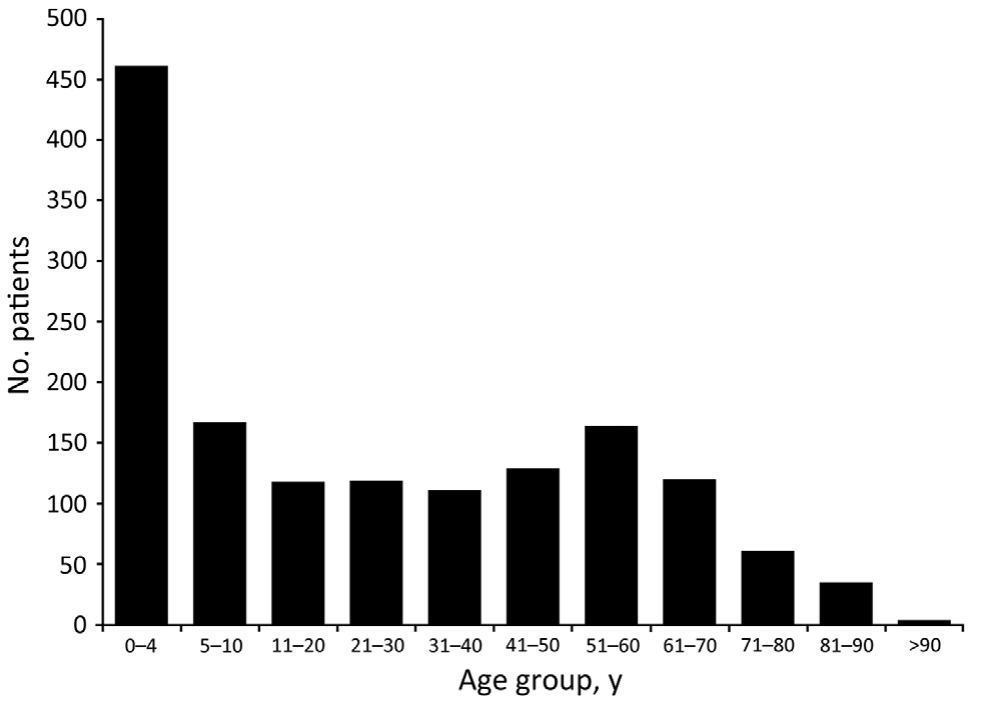
Number of patients in reported live poultry–associated salmonellosis outbreaks, by age group, United States 1991–2014.

Montevideo was the serotype identified in 36% (19/53) of LPAS outbreaks, making it the most common *Salmonella* serotype reported. Among the *Salmonella* strains associated with LPAS outbreaks, 62% (38/61) were serogroup C_1_; serogroup B accounted for 16% (10/61), and serogroup C_2_ accounted for 13% (8/61). Serogroups C_3_, D_1_, and R were also reported. Additionally, 4 outbreaks consisted of multiple *Salmonella* serotypes. In the outbreaks with available information, 54% (1,026/1,898) of case-patients were male, and 46% (872/1,898) were female. Median case-patient age was 9 years (range <1 to 92 years); 31% (467/1,488) of case-patients were <5 years of age, and 42% (628/1488) were <10 years of age (Figure 2).

A total of 62% (511/822) of case-patients reported exposure to baby poultry (Table 2). Chick exposure was reported by 85% (434/511) of case-patients and duckling exposure by 38% (195/511). Among case-patients exposed to baby poultry, 62% reported exposure to only chicks (316/511), 15% (77/511) exposure to only ducklings, and 22% (118/511) exposure to both chicks and ducklings. Approximately 23% (117/582) of respondents reported contact with adult poultry. Among all outbreaks, the median time between purchase of poultry and illness onset was 17 days (range 1–672 days). Approximately 66% of case-patients reported <30 days between obtaining poultry and illness onset. However, 7% of case-patients reported >60 days between obtaining poultry and illness onset.

**Table 2:** Selected exposure characteristics of patients in 21 multistate live poultry–associated salmonellosis outbreaks, United States, 2008–2013*

Characteristic	No. (%) patients
Type of poultry, n = 822	
Adult poultry	161 (20)
Baby poultry	511 (62)
Chicks only	316 (62)
Ducklings only	77 (15)
Chicks and ducklings	118 (23)
Contact location, n = 413	
Indoors	188 (46)
Living room	41 (11)
Basement	57 (15)
Kitchen	22 (12)
Bedroom	18 (10)
Bathroom	18 (10)
Utility room/laundry room	17 (9)
Other indoor	44 (23)
Type of contact, n = 400	
Touched	303 (76)
Held/snuggled	196 (49)
Kissed	53 (13)

*n values indicate number of respondents for each question.

Among respondents with baby poultry exposure, 74% (276/373) reported that exposure occurred at the home. Approximately 76% (303/400) of respondents reported touching baby birds, 61% (227/373) reported touching the cage/coop of the baby birds, 49% (196/400) reported snuggling baby birds, and 13% (53/400) reported kissing baby birds.

Nearly 46% (188/413) of respondents reported keeping poultry inside the house. Of these, 22% (41/188) reported keeping live poultry in the living room, 12% (22/188) in the kitchen, 10% (18/188) in a bedroom, and 10% (18/188) in a bathroom. Approximately 52% of respondents reported owning poultry for <1 year. When asked if they were aware of a connection between poultry contact and *Salmonella*, 58% (167/290) of respondents reported that they were aware of the risk.

## Discussion

The number of LPAS outbreaks reported annually has increased substantially in recent years. Because only a small proportion of *Salmonella* infections are diagnosed and reported to public health departments, the actual number of illnesses in these outbreaks might be much larger with an estimated 29 additional infections going unreported for every reported case (*2*). These outbreaks are not only happening with increased frequency but are also generally affecting more persons. In addition, 62% of case-patients reported contact with baby chicks or ducklings, and 45% were <10 years of age. This finding is possibly attributable to the fact that children’s immune systems are not fully developed and that young children typically have poor hand hygiene practices. Most contact occurred at the patients’ home, and high-risk behaviors included keeping poultry inside the house and having close contact, such as holding, snuggling, or kissing poultry. These findings highlight the need for additional consumer education, especially on the risk for illness in children, the necessity for keeping live poultry outside of the home, and the recommendation to wash hands after coming in contact with live poultry.

Instead of being sold as novelty pets around the Easter holiday, chicks, ducklings, goslings, and turkey poults are now additionally being sold for backyard flocks; these birds can be purchased at agricultural feed stores across the United States. The practice of keeping backyard flocks of live poultry has gained popularity during the past decade (*11*). This increase is attributable to various reasons, including growing interest in local and organic food production, animal welfare concerns, environmental concerns, the desire to provide a learning experience for children, and the perception that local eggs are healthier and of better quality than store-bought eggs (*34*). In addition, backyard flocks are becoming increasingly common in urban and suburban areas (*11*). The fact that half of respondents to the live poultry questionnaire owned poultry for <1 year could signify that new owners might be unfamiliar with appropriate husbandry practices.

Most poultry sold for backyard flocks are produced by a core group of ≈20 mail-order hatcheries. These hatcheries sell more than 50 million chicks annually, and most distribute chicks nationwide (*20*). Distribution of baby poultry occurs through the US Postal Service, by which a small proportion are mailed directly to owners, whereas most baby poultry are sold to agricultural feed stores. Baby poultry are shipped in cardboard boxes that can contain 120 chicks, 60 ducklings, 32 goslings, or 80 turkey poults. One box may contain multiple species, and shipment can provide ample opportunity for cross-contamination. Increased shedding of *Salmonella* can occur when poultry are subjected to stressful conditions, such as transportation through the mail (*6*). The nationwide distribution as well as the opportunity for cross-contamination might help to explain the multistate distribution of outbreaks.

The serotypes identified in LPAS outbreaks are different from *Salmonella* serotypes, such as Enteritidis and Heidelberg, that are traditionally associated with foodborne poultry outbreaks (*35*,*36*). This finding might be attributable in part to differences in poultry that originate from mail-order hatcheries and commercial poultry hatcheries. Mail-order hatcheries typically operate on a much smaller scale, with more species and breeds of poultry sourced from breeding stock within their own farm or eggs from other mail-order hatcheries, which could explain the diversity of *Salmonella* serotypes identified in these outbreaks. In comparison, commercial poultry operations are typically larger scale, closed operations with 1 species and fewer breeds of bird on site (*37*).

The seasonality of these outbreaks might be attributable to the fact that most agricultural feed stores sell large numbers of chicks during spring or fall promotional events or “chick days.” These events provide additional opportunity for cross-contamination in the stores because of the increase in volume of chicks during these events. In addition to the increase in volume of chicks being sold in the spring, some households might keep chicks inside the home because of concerns that the chicks will not do well in cold weather.

Poultry can appear healthy and still shed *Salmonella* bacteria intermittently for extended periods of time (*38*). This intermittent shedding could contribute to the fact that some case-patients reported illness onset >1 year after poultry purchase. In addition, intermittent shedding could partially explain why recent outbreaks have been of a longer duration, some lasting up to 12 months.

The findings of this investigation are subject to several limitations. Smaller, single-state outbreaks might have been missed if they were not reported to National Outbreak Reporting System or not documented elsewhere. Additional outbreaks might have been missed if they were not detected by PulseNet or if, during the course of the investigation, public health practitioners did not ask case-patients about exposure to live poultry. Finally, results of the supplemental poultry questionnaires were only available for multistate outbreaks that occurred in 2008 or later. Case-patients from earlier outbreaks might have had different characteristics and had different types of poultry exposure in comparison to case-patients from the more recent outbreaks. Because our study relied on aggregated outbreak data, we could not calculate the relative magnitude of risk for different handling practices; therefore, we are unable to state which practices contribute the most to *Salmonella* transmission from live poultry to humans.

This review highlights the need for an integrated One Health response to LPAS outbreaks. One Health is defined as the collaborative effort of experts in multiple disciplines, including healthcare professionals, veterinarians, epidemiologists, and environmental scientists, working to attain optimal health for humans, animals, and the environment (*7*). Prevention and control efforts for LPAS outbreaks include interventions that target hatcheries, agricultural feed stores, health professionals, and consumers. Detailed recommendations for a comprehensive One Health prevention approach are available (*9*).

**Figure 3:**
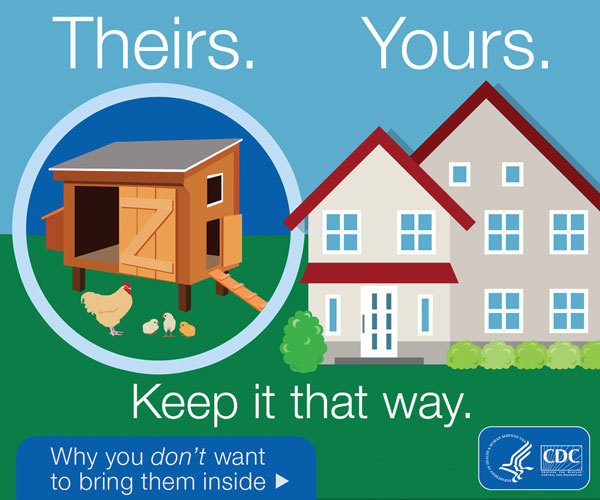
A “Tips to Stay Healthy around Backyard Poultry Flocks” web graphic produced by the Centers for Disease Control and Prevention.

To prevent future outbreaks, the general public needs to be educated about the risk for LPAS. Persons need to be aware that healthy poultry can shed *Salmonella* intermittently, that persons need to wash their hands after contact with live poultry, that young children are at an increased risk for salmonellosis, and that poultry should never be allowed inside the house. Mail-order hatcheries, agricultural feed stores, public health officials, local and federal departments of agriculture, pediatricians, and veterinarians can all help to spread awareness about the association between live poultry and *Salmonella* infections. CDC has developed various educational resources that mail-order hatchery Web sites can link to (Figure 3). Posters and additional educational material can be displayed at points of sale (*39*). CDC has participated in a series of online consumer educational webinars with the US Department of Agriculture and other poultry interest groups (*40*). Healthcare providers can talk to parents about the risk for zoonotic *Salmonella* in children, especially if high-risk pets are in the home (*41*,*42*). The Journal of the American Veterinary Medical Association recently reported on the increased need for veterinarians who are willing to treat backyard poultry (*43*). Veterinarians have a unique opportunity to educate poultry owners about *Salmonella* prevention and control strategies (*9*).

Poultry are acquiring a new position in many households. Instead of being treated as production animals, they are increasingly being considered household pets. However, recurring LPAS outbreaks highlight the need for strategies to prevent human illnesses associated with live poultry contact through a comprehensive One Health approach involving human, animal, and environmental health.
